# Ivermectin Inhibits Zika Virus Replication in Vitro But Does Not Prevent Zika Virus Infection in Rhesus Macaques (*Macaca mulatta*)

**DOI:** 10.4269/ajtmh.24-0183

**Published:** 2024-12-17

**Authors:** Thomas S. Cotrone, Kevin Kobylinski, Alongkot Ponlawat, Rawiwan Im-Erbsin, Piyanate Sunyakumthorn, Pattaraporn Vanachayangkul, Yongyuth Poolpanichupatam, Jindarat Lohachanakul, Chonticha Klungthong, Aaron Farmer, Stefan Fernandez, Taweewun Hunsawong

**Affiliations:** ^1^ Department of Virology, Walter Reed Army Institute of Research-Armed Forces Research Institute of Medical Science (WRAIR-AFRIMS), Bangkok, Thailand;; ^2^ Department of Entomology, Walter Reed Army Institute of Research-Armed Forces Research Institute of Medical Science (WRAIR-AFRIMS), Bangkok, Thailand;; ^3^ Department of Veterinary Medicine, Walter Reed Army Institute of Research-Armed Forces Research Institute of Medical Science (WRAIR-AFRIMS), Bangkok, Thailand;; ^4^ Department of Bacterial and Parasitic Diseases, Walter Reed Army Institute of Research-Armed Forces Research Institute of Medical Science (WRAIR-AFRIMS), Bangkok, Thailand

## Abstract

Zika virus (ZIKV) outbreaks occur sporadically in tropical and subtropical regions. At present, there are no licensed vaccines or specific treatments available for ZIKV. Ivermectin is approved for use in humans as an antiparasitic drug. In this study, we conducted in vitro cell culture and in vivo experiments in rhesus macaque hosts and *Aedes aegypti* vectors to investigate the potential of ivermectin as an inhibitor of ZIKV infection. In LLC-MK_2_ mammalian cells, ivermectin inhibited ZIKV growth in vitro with 50% inhibitory concentration (IC_50_) values in the ranges of 7.4–21.3 µM and 4.0–11.6 µM for African and Asian genotypes, respectively. In C6/36 mosquito cells, ivermectin inhibited ZIKV growth in vitro with IC_50_ values in the ranges of 10.1–17.4 µM and 8.0–15.6 µM for the African and Asian genotypes, respectively. Despite these in vitro results, high-dose ivermectin prophylaxis (1.2 mg/kg for 3 consecutive days) failed to prevent ZIKV infection in rhesus macaque and did not alter ZIKV IgM antibody production. The secondary transfer of ivermectin from nonhuman primate blood to mosquito vectors at 3 days post-ZIKV inoculation and after the last dose of ivermectin administration showed no reduction in ZIKV replication in mosquitoes. However, mosquito survival rates were significantly (*P* <0.0001) lower after exposure to ivermectin, thereby potentially impacting ZIKV transmission through increased vector mortality. However, further investigation is needed to determine dosing regimens that may realize these effects in vivo.

## INTRODUCTION

The re-emergence of Zika virus (ZIKV) poses a significant global health threat as the geographic distribution of this virus continues to expand globally. Several ZIKV vaccines have been developed, but none of them have been licensed.^1^^–^^3^ The current treatment of ZIKV infection is limited to symptom management. An antiviral drug that directly targets ZIKV would be a useful tool for disease control and management. ZIKV was first isolated from a rhesus macaque in the Zika Forest, Uganda in 1947.^4^ The range of ZIKV has expanded, emerging in Micronesia (2007); French Polynesia, New Caledonia, Easter Island, and the Cook Islands (2013)^5^^,^^6^; Philippines (2012)^7^; Thailand (2012–2014)^8^; and most recently, the large epidemics in the Americas and the Caribbean (2015).^9^ ZIKV is a member of the *Orthoflavivirus* genus,^10^ Flaviviridae family, which transmits to humans by the bite of *Aedes* mosquitoes as a primary transmission mode. ZIKV can also spread directly from person to person through sexual contact^11^^,^^12^ and vertically from mother to fetus.^13^ The clinical spectrum of ZIKV infection ranges from asymptomatic infection to severe disease, similar to dengue virus (DENV) and chikungunya virus (CHIKV) infections.^14^ However, unlike DENV and CHIKV, ZIKV is associated with severe neurological complications, including microcephaly in fetuses^15^^,^^16^ and Guillain–Barre syndrome in adults infected with ZIKV.^17^ Consequently, ZIKV poses a unique and significant public health risk to pregnant women.^18^^–^^20^

Ivermectin is a safe antiparasitic drug that is commonly used to treat and control human lymphatic filariasis, onchocercisasis, *Strongyloides*, and scabies.^21^^,^^22^ It has been approved for use in humans at concentrations of 150–200 µg/kg (171–228 mM/kg). After administration of 150 µg/kg (171 mM/kg) of drug, the concentration of ivermectin in human plasma ranges from 9 to 75 ng/mL (10.3–85.5 pM) and rapidly declines in the following days.^23^ Ivermectin has been studied for its insecticidal effects on *Anopheles* mosquitoes, which are malaria vectors. Research indicates that when humans or animals are treated with ivermectin, it can transfer to mosquitoes through blood meals, impacting their survival and reproduction in a dose-dependent manner.^24^ Recent studies have shown that ivermectin can also exhibit potent antiviral effects in vitro, such as inhibition of ZIKV and CHIKV replication.^25^^,^^26^ Ivermectin has been shown to inhibit NS3 helicase activity of flaviviruses, such as yellow fever virus, DENV, and West Nile virus in vitro.^27^ NS3 is essential to flavivirus replication because of its protease, helicase, adenosine triphosphatase, and RNA 5′ triphosphatase functions.^28^ Taken together, these factors could prove relevant to ZIKV replication inhibition because DENV NS3 shares 67% and 50% of its amino acid identity with NS3 of ZIKV and yellow fever virus, respectively.^29^ Moreover, ivermectin could interfere with both HIV-1 and DENV replication by inhibiting the function of importin *α*/*β*, which plays a role in recognition and import of viral nucleoprotein into the nucleus of host cells during the virus replication cycle.^30^ These mechanistic links between ivermectin and viral replication have been shown to translate into relevant viral inhibition in vitro. For example, a compound screening of 3,000 potential targets found ivermectin as one of the three most potent antiviral drugs against CHIKV replication.^25^

Therefore, we investigated the ability of ivermectin to inhibit ZIKV replication in mammalian and mosquito cell lines. We also investigated the prophylactic properties of ivermectin in a rhesus macaque model of ZIKV (Asian genotype, Puerto Rico strain) infection and its effect on subsequent viral infectivity to the *Aedes aegypti* mosquito vector.

## MATERIALS AND METHODS

### Cell lines.

LLC-MK_2_ cells (rhesus macaque kidney epithelial cells) and C6/36 cells (*Aedes albopictus*-derived larval cells) were obtained from American Type Culture Collection, and they served as the mammalian and mosquito cell lines, respectively, for this study. LLC-MK_2_ cells were grown in Minimum Essential Medium (MEM; Invitrogen, Waltham, MA) medium containing 10% heat-inactivated fetal bovine serum (HIFBS; Invitrogen). C6/36 cells were grown in Roswell Park Memorial Institute (Invitrogen) containing 10% HIFBS. Both LLC-MK_2_ and C6/36 cell lines were maintained at 35°C in a 5% CO_2_ incubator.

### Viruses.

ZIKV African genotype (MR766; obtained from the American Type Culture Collection VR84: C6/36-4; ATCC, Manassas, VA), ZIKV Asian genotype SV127/14 (Thai isolated strain C6/36-3; GenBank: KU681081; GenBank, Bethesda, MD), and the Puerto Rico-isolated strain PRVABC59 (obtained from the Viral Disease Branch, Walter Reed Army Institute of Research, Vero-2, C6/36-2; GenBank: KU501215) were propagated in C6/36 cells to generate titers of 10^6^ Plaque forming unit (PFU)/mL for infection. Viral genome sequencing was performed to confirm viral lineage before using. Multiple aliquots of the same lot of viruses were kept at −80°C until used.

### Mosquitoes.

*Ae. aegypti* (Armed Forces Research Institute of Medical Sciences [AFRIMS] strain) mosquitoes were originally collected from villages in Bangkok, Thailand in August 1992 and maintained in the insectary at the Department of Entomology, AFRIMS. The number of generations of mosquitos is over 300. They were reared and maintained under laboratory conditions of 25°C ± 1°C and 80% ± 10% relative humidity with a photoperiod of 12:12 hours of light per dark cycle in the insectary section of the Department of Entomology, AFRIMS. Adults were provided ad libitum 10% sucrose solution for energy and hydration until the start of the experiment. In preparation for feeding on nonhuman primates (NHPs), *Ae. aegypti* females (3–5 days old) were placed into the paper cups (30 mosquitos at a time) and subsequently, starved for 12 hours before feeding.

### Rhesus macaques.

Walter Reed Army Institute of Research-Armed Forces Research Institute of Medical Science colony-born rhesus macaques of Indian origin were used in this study. Twelve rhesus macaques (*Macaca mulatta*; four males and eight females) that met the inclusion criteria for the study (including age [5–9 years old]; body weight [5–12 kg]; negative testing for both simian retroviruses and simian herpes B virus; and hemagglutination assay^31^^,^^32^-confirmed seronegative for DENV-1 to DENV-4, Japanese encephalitis virus, and ZIKV) were selected for this study.

### Ivermectin.

For in vitro experiments, a powdered formulation of ivermectin was obtained from Sigma-Aldrich and dissolved in dimethylsulfoxide (DMSO; Sigma-Aldrich, Burlington, MA) to a final concentration of 57 mM. Stock ivermectin solution was stored at −20°C. Freshly prepared drug was used for each independent cell treatment. For in vivo experiments, Sparmectin-E^®^ (ivermectin 10 mg/mL; lot 191211) was used. Sparmectin-E is a water-soluble formulation of ivermectin developed for oral use in horses. Doses were prepared daily by diluting the compound with sterile water (lot 2009096).

### Virus infectivity assay.

LLC-MK_2_ cells were seeded at a density of 2 × 10^4^ cells/well and C6/36 cells were seeded at a density of 3 × 10^4^ cells/well in 96-well plates. Cultures were incubated for 2 days to reach 100% cell confluence. On the day of the experiment, cells were pretreated with ivermectin solution (20, 10, or 1 µM in DMSO vehicle), 0.04% DMSO in cell culture media, or cell culture media alone for 4 hours at 35°C in a 5% CO_2_ incubator. Without washing, drug-treated plates were separated into two groups and infected with either ZIKV African genotype (MR766) or ZIKV Asian genotype (SV127/14) at an multiplicity of infection (MOI) of two. Cultures were subsequently incubated for 2 hours at 35°C in a 5% CO_2_ incubator. After incubation, cells were washed twice with media before adding new media without the drug. During the next 48 hours, cells were incubated at 35°C, and tissue culture supernatant was harvested at 3, 6, 18, 24, or 48 hours postinfection. The number of infectious viral particles in each sample was quantified with a plaque assay.

### Virus yield and cytopathic effect reduction assay.

LLC-MK_2_ and C6/36 cells were seeded at a density of 2 × 10^4^ cells/well and 3 × 10^4^ cells/well in 96-well plate, respectively. Cells were treated with various concentrations of ivermectin solution (20, 10, or 1 µM in DMSO vehicle), 0.04% DMSO in cell culture media, or cell culture media alone. Immediately after this treatment, cells were separated into two groups that were infected with either ZIKV MR766 or ZIKV SV127/14 at an MOI of 0.1 PFU/cell. After incubation for 2 hours at 35°C in 5% CO_2_, cells were washed three times with cell culture media. Fresh medium containing ivermectin was added to each culture and subsequently incubated for 4 days. Cytopathic effect (CPE) formation was observed daily using light microscopy. Cell supernatant was harvested on day 4 postinfection, and infectious virus yield reduction was determined by plaque assay.

### Plaque assay.

Virus titer in the cell supernatant was quantified by plaque assay using LLC-MK_2_ cells. Samples were 10-fold serial diluted (1:10 to 1:10,000) with tissue culture media: MEM (Invitrogen) supplemented with 10% HIFBS (Invitrogen); 100 µL of diluted sample was added to LLC-MK_2_ cell monolayers. After incubation for 1 hour at room temperature, the inoculum was removed. The first overlay, 1.8% low-melting point agarose gel, was added before incubation at 35°C at 5% CO_2_ for 4 days. Plaque formation was visualized by applying a second overlay containing 4% neutral red.

### Rhesus macaque trial.

Twelve macaques were randomly assigned 1:1 to either a control (sham-treated) group or a treatment (ivermectin-treated) group. For the treatment group, NHPs were oral administered ivermectin or sham (water) control through a nasogastric tube at 1.2 mg/kg of ivermectin daily for 3 consecutive days. Whole blood was collected from each NHP at 0, 4, 24, 28, 48, 52, and 120 hours to determine ivermectin plasma concentrations through subsequent pharmacokinetic analysis. Four hours after administration of the final ivermectin dose, all NHPs were subcutaneously injected in the upper medial right arm with 10^6^ PFU (1 mL of diluted virus in phosphate buffer saline (PBS) [Invitrogen] per NHP) of ZIKV Puerto Rico strain (PRVABC59). For each animal, blood was collected on days 0, 1, 2, 3, 4, 5, 6, 7, and 14 postinfection to evaluate viremia and anti-ZIKV IgM antibody production.

### Mosquito infectivity and survival evaluation.

To investigate the effect of ivermectin secondary administration on mosquito vectors through blood feeding on NHPs and its impact on viral transmission, female *Ae. aegypti* (AFRIMS strain, age 3–5 days old) were randomly assigned to one of two groups of monkeys (540 mosquitoes per group, three batches of 30 mosquitoes per monkey; i.e., 90 mosquitos per monkey). Mosquitoes were allowed to feed on all NHPs in their assigned groups (*n* = 6 monkeys per group) at 3 days after ZIKV inoculation and ivermectin administration. Blood-engorged *Ae. aegypti* were then maintained in an incubator with 12:12 light-to-dark hours at 28°C ± 1°C and 80% ± 10% humidity with access to 10% sugar ad libitum. After feeding, one of the three batches of mosquitoes was harvested on days 0 (6 hours), 7, and 14 postblood meal for each NHP. During harvesting of each batch, the total number of dead mosquitoes was counted before the surviving mosquitoes were euthanized. Midguts, legs, and salivary glands were dissected and harvested to determine the infection, dissemination, and transmission rates by quantitative reverse transcription polymerase chain reaction (RT-PCR).

### Quantitative RT-PCR for ZIKV RNA determination.

For NHP whole-blood samples, serum was separated by centrifugation, and ZIKV RNA was extracted from 140 µL of serum using the QIAamp Viral RNA Mini Kit (QIAGEN, Hilden, Germany). For mosquito tissues, head/thorax, abdomen, and leg tissues were homogenized, and ZIKV RNA was extracted using the RNeasy Mini Kit (QIAGEN) per the manufacturer’s instruction. Positive/negative extraction controls and positive/negative controls for quantitative RT-PCR were included in each experiment. ZIKV real-time quantitative RT-PCR was performed using a method modified from Lanciotti et al.^5^ Two primer/probe sets were used: 1) ZIKV 1086 forward, ZIKV 1162c reverse primers, and ZIKV 1107-FAM probe (Colombo et al. 2018) and 2) ZIKV 4434 forward, ZIKV 4524c reverse primers, and ZIKV 4479c-FAM probe (Waggoner and Pinsky 2016). All assays were performed using the SuperScript III Platinum One-Step Quantitative RT-PCR Kit (Invitrogen) per the manufacturer’s instructions using the Applied Biosystems 7500 Fast Real-Time PCR Systems (Thermo Fisher Scientific, Waltham, MA). Limit of quantification of the assay was 5 genome equivalents/mL.

### ZIKV IgM enzyme-linked immunosorbent assay.

An in-house capture in-house ZIKV IgM enzyme-linked immunosorbent assay was used to detect anti-ZIKV IgM antibody in the serum of NHPs.^33^ Briefly, flat-bottomed microplates were coated with 100 µL/well of goat antihuman IgM (KPL, Gaithersburg, MD) in 0.018 M carbonate buffer, pH 9.0. After overnight incubation at 4°C, plates were washed with phosphate buffer saline, pH 7.4, containing 0.5% Tween 20 (PBS-T). After washing, 50 µL/well of a 1:100 dilution of test serum, negative control (NC), weak positive control (WPC, and strong positive control in PBS were added to separate wells of the plates. Plates were incubated overnight at 4°C and then washed with PBS-T. After washing, 50 µL/well of sucrose acetone-extracted suckling mouse brain ZIKV (MR766; Uganda 1947) antigen (50–100 hemagglutination units) was added to the microplate. After incubation for 2 hours at 37°C, 30 µL/well of human antiflavivirus IgG–horseradish peroxidase conjugate was added to each well, and plates were incubated for 1 hour at 37°C. After washing, 100 µL/well of 3,3′,5,5′-Tetramethylbenzidine (TMB) substrate (KPL) was added before incubating for another 30 minutes The reaction was stopped by adding 0.2 M sulfuric acid to each well (50 µL/well). Optical absorbance at optical density 450 was measured and calculated for enzyme immunoassay (EIA) units. Enzyme immunoassay units of tested serum are equal to 100 × [(OD_Test_ − OD_NC_)/(OD_WPC_ − OD_NC_)]. A threshold of EIA units of IgM ≥40 was used as a positive cutoff value.

### Ivermectin quantification by liquid chromatography–mass spectrometry.

Blood was collected in heparinized sodium Vacutainer tubes and centrifuged at 2,500 rpm for 20 minutes The supernatant (plasma) was subsequently transferred and kept at –80°C until analysis was performed. Plasma was separated into two tubes with 200–400 µL in each tube. Ivermectin was extracted using the protein precipitation method with 2:1 acetonitrile (with internal standard)-to-plasma volume, vortex mixed for 1 minute, and then centrifuged at 10,000 rpm for 10 minutes A total of 200 µL of supernatant fluid was filtered through a 0.22-µm polytetrafluoroethylene membrane before injection into a ultraperformance liquid chromatography (UPLC) system.

The liquid chromatography–mass spectrometry was performed on an Acquity UPLC system equipped with a Xevo G2-XS quadrupole time of flight mass spectrometer (Waters Corp., Milford, MA). A Waters Acquity UPLC ethylene-bridged hybrid C18 column (50 × 2.1 mm, 1.7-µm particle size) with a precolumn of the same material was used to separate the compounds. The gradient mobile phase used for analysis of ivermectin was 5 mM ammonium formate and 0.1% formic acid in water and methanol with a column temperature of 40°C and a flow rate at 0.4 mL/minute The total run time was 7 minutes, and the injection volume was 5 µL. Mass spectrometry was set in the positive electrospray ionization mode with multiple reaction monitoring. Instrument parameters included capillary voltage of 3.5 kV and source and desolvation temperatures of 150°C and 400°C, respectively. The nitrogen generator was set at 120 lb/in^2^ to generate cone and desolvation gas flow rates of 50 and 800 L/hour, respectively. The mass transitions were observed at *m/z* of 892.77–569.50 and 894.79–571.52 for ivermectin and ivermectin-D2, respectively. Masslynx software (Waters Corp.) was used for quantification.^34^

### Pharmacokinetic modeling and simulation.

Previously generated noncompartmental analysis pharmacokinetic parameters^34^ were used as parameter estimates for compartment modeling with the pharmacokinetic data collected in this trial. Observed ivermectin concentrations were best described by one-compartment analysis with first-order absorption and first-order elimination.

## STATISTICAL ANALYSES

Statistical analysis of the inhibitory effect of ivermectin on ZIKV infection in both in vitro and in vivo systems was performed using SPSS program v. 26 (IBM Corp., Armonk, NY) and GraphPad Prism v. 9.0.0 (Boston, MA). The 50% inhibitory concentration (IC_50_) value of ivermectin was determined as the concentration at which the viral titer was reduced by 50% as compared with virus control wells using a dose–response curve analysis (GraphPad Prism). The Mann–Whitney *U* test/Kruskal–Wallis analysis was used to compare the differences of virus titers in the presence and absence of ivermectin. The log-rank (Mantel–Cox) test was used to compare the survival of mosquitoes between treatment and control groups. Differences with *P*-values of less than 0.05 were considered statistically significant.

## RESULTS

### Effect of ivermectin on ZIKV replication in vitro.

**Figure 1.**
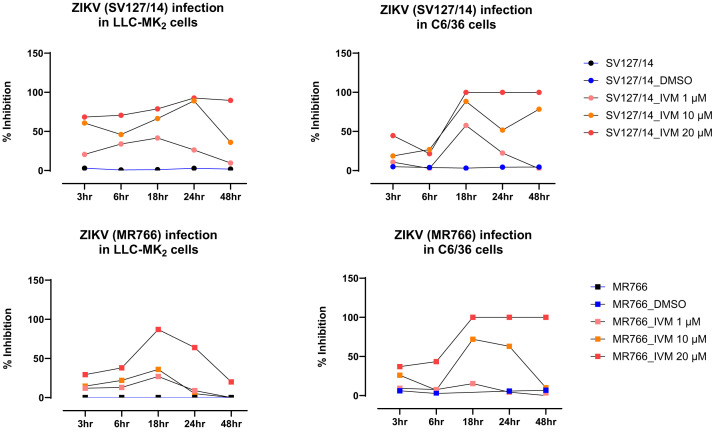
The effect of ivermectin on the ZIKV growth curve in the mammalian cell line (LLC-MK_2_) and the mosquito cell line (C6/36). The upper panels show ZIKV (Asian genotype: SV127/14) growth curves in LLC-MK_2_ and C6/36 cell lines. The lower panels show ZIKV (African genotype: MR766) growth curves in LLC-MK_2_ and C6/36 cell lines. DMSO = dimethylsulfoxide; hr = hour; IVM = ivermectin; ZIKV = Zika virus.

We conducted both virus infectivity and virus yield/CPE reduction assays to identify the effect of ivermectin on early ZIKV viral replication dynamics and overall virus production on late-stage infection outcomes, respectively. Additionally, the virus yield and CPE reduction assay typically includes determination of IC_50_ values, providing a quantitative measure of ivermectin’s potency in reducing viral replication. Before testing the in vitro antiviral effects of ivermectin on ZIKV, the toxic effects of ivermectin on both mammalian (LLC-MK_2_) and insect (C6/36) cell line growth curves were assessed at various concentrations. An ivermectin concentration of 20 µM was identified as the maximum concentration that had no effect on growth curves of either cell line (Supplemental Figure 1). Subsequently, 20, 10, and 1 µM concentrations of ivermectin were selected for further testing to evaluate the antiviral effects of ivermectin on ZIKV infection. At 18 hours postinoculation, ivermectin treatment significantly reduced replication of both the African (MR766) and Asian (SV127/14) genotypes of ZIKV in LLC-MK_2_ and C6/36 cell lines in a dose-dependent manner (*P* <0.0001) compared with that seen in the control and DMSO vehicle-treated cells (Figure 1).

Against the Asian ZIKV genotype, both 10 and 20 µM concentrations of ivermectin treatment resulted in a 50% or higher reduction of viral growth in both LLC-MK_2_ and C6/36 cells. However, 20 µM ivermectin was the only concentration that maintained viral inhibition above 50% in both cell lines at 48 hours postinfection (i.e., the end of the experiment). Treatment with 10 µM ivermectin maintained inhibition of the Asian ZIKV strain in mammalian cells at 48 hours postinfection, but it failed to do so in mosquito cells. Treatment with 1 µM ivermectin failed to maintain 50% viral inhibition by 24 hours postinfection in both cell lines.

Conversely, against the African ZIKV genotype, only 20 µM ivermectin achieved 50% or higher inhibition of viral growth in both cell lines. Treatment with 10 µM ivermectin induced and maintained viral inhibition above 50% in mosquito cells at 48 hours postinfection; however, it never reached 50% inhibition in the mammalian cell line at any time point. For both cell lines, 1 µM ivermectin failed to reach viral inhibition above 30%.

We evaluated the IC_50_ values of ivermectin for ZIKV infections in mammalian (LLC-MK_2_) and mosquito (C6/36) cell lines. For LLC-MK_2_ cells, the IC_50_ (95% CI) values for the ZIKV African genotype and the ZIKV Asian genotype were 10.5 µM (7.4–21.3 *µ*M) and 6.6 µM (4.0–11.6 *µ*M), respectively. In C6/36 cells, the IC_50_ (95% CI) values were shown as 13.4 µM (10.1–17.4 *µ*M) and 11.2 µM (8.0–15.6 µM) for the ZIKV African genotype and the ZIKV Asian genotype, respectively (Table 1). The lower IC_50_ values of ivermectin for the ZIKV Asian genotype in both mammalian and mosquito cell line infections indicate that the ZIKV Asian genotype was more sensitive to ivermectin treatment than the ZIKV African genotype.

**Table 1:** The half-maximal inhibitory concentration of ivermectin on the inhibition ZIKV-infected mammalian cell line (LLC-MK_2_) and the mosquito cell line (C6/36)

Cell Lines	IC_50_ (*µ*M; 95% CI)	
	ZIKV (MR766) African Genotype	ZIKV (SV127/14) Asian Genotype
LLC-MK_2_ (mammalian)	10.5 (7.4–21.3)	6.6 (4.0–11.6)
C6/36 (mosquito)	13.4 (10.1–17.4)	11.2 (8.0–15.6)

IC_50_ = 50% inhibitory concentration; ZIKV = Zika virus.

### Ivermectin pharmacokinetics in macaques.

To validate that ivermectin administration was successful, pharmacokinetic analysis of ivermectin was performed using serum samples collected from each ivermectin-treated NHP at 0, 4, 24, 48, 52, 72, and 120 hours after administration of the first ivermectin dose (1.2 mg/kg). This analysis revealed that the range of ivermectin concentrations in serum samples was between 538.7 and 1,307.9 ng/mL (0.616–1.495 µM) with a median (interquartile range) of 785.5 ng/mL (0.898 µM) at the time of ZIKV inoculation (i.e., 52 hours after the first ivermectin dose). At 72 hours after NHPs received the last dose of administration, which coincided with the time for mosquito feeding on NHP blood, the concentration of ivermectin in the blood was measured at 194.8 ng/mL with a range from 111.8 to 277.8 ng/mL.

### Effect of ivermectin treatment on ZIKV viremia levels in NHPs.

**Figure 2.**
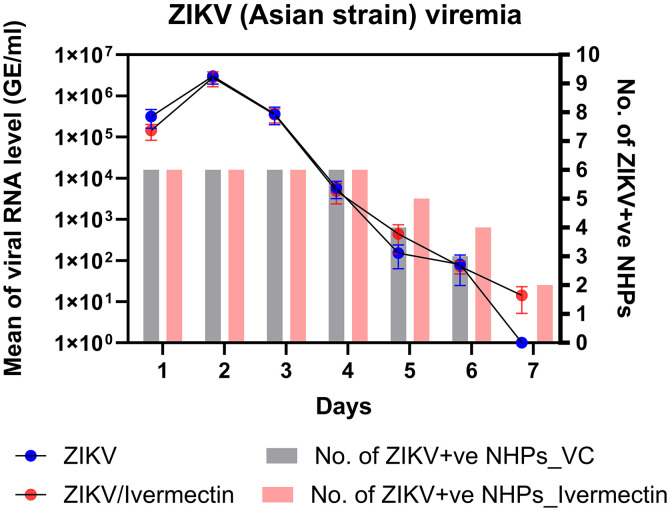
ZIKV viremia in NHPs. ZIKV RNA levels in the serum of ZIKV infection and ivermectin treatment groups were detected by quantitative reverse transcription polymerase chain reaction. The left *y* axis represents the mean of viral RNA levels in GE per milliliter units, and the right *y* axis represents the positive rate of NHPs in each day. GE = genomic equivalent; NHP = nonhuman primate; VC = virus control; ZIKV+ve = Zika virus positive.

Serum was collected daily from ZIKV-infected NHPs between 1 and 7 days postinfection (DPI) to evaluate the effect of ivermectin on ZIKV viremia (Figure 2). Quantitative RT-PCR revealed that all NHPs had detectable ZIKV messenger RNA (mRNA) levels in serum beginning on 1 DPI. Within both control and ivermectin-treated groups, a significant effect of time on ZIKV mRNA was observed. Analysis of within-group effects of DPI on ZIKV mRNA revealed a significant increase in viral mRNA between 1 and 2 DPI (*P* = 0.0317). This was followed by a significant decrease in viral mRNA between 2 and 3 DPI (*P* = 0.0317) and a continual decline in viral mRNA until the end of the experiment at 7 DPI. However, analysis of the effects of ivermectin treatment on ZIKV mRNA (i.e., between-group effects) revealed no significant differences in viral mRNA levels at any time point. These findings were consistent with the plaque assay results, which failed to show any significant effect (*P* = 0.9015) of ivermectin treatment on viral particle load in the serum compared with that of untreated controls.

Ivermectin treatment failed to significantly reduce the number of ZIKV-positive macaques at 1–6 DPI compared with controls (Figure 2). On day 7 (i.e., the last day of the study), the number of ZIKV-positive animals had decreased in both the ivermectin and sham-treated groups; however, a between-group comparison at this time point revealed a significantly greater reduction in the number of positive animals within the control group compared with that of the ivermectin-treated group. Similarly, a between-group comparison of plaque assay results showed that there were no significant effects (*P* = 0.7960) of ivermectin treatment on the number of NHPs that were ZIKV positive at any time point during the study.

### Effect of ivermectin treatment on anti-ZIKV IgM antibody production in NHPs.

**Figure 3.**
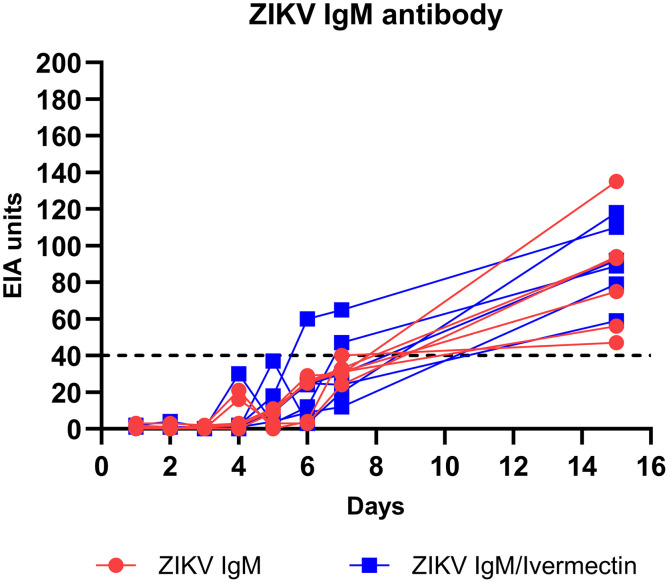
Anti-ZIKV IgM antibody production in nonhuman primates. Anti-ZIKV IgM antibody production in the serum of ZIKV infection and ivermectin treatment groups was detected by an in-house anti-ZIKV IgM capture enzyme-linked immunosorbent assay. The dashed black line indicates a positive cutoff (≥40 EIA units) for the IgM antibody. EIA = enzyme immunoassay; ZIKV = Zika virus.

Anti-ZIKV IgM antibody production was measured in serum collected from NHPs 1–7 DPI and 15 DPI (Figure 3). ZIKV IgM antibodies were first detected 4 DPI in both the sham-treated group (*n* = 2; 21 and 16 EIA units) and the ivermectin-treated group (*n* = 1; 30 EIA units). At 7 DPI, ZIKV IgM antibodies were detected in all NHPs of both groups, with the means (95% CIs) at 32 EIA units (26–37 EIA units) and 33 EIA units (12–54 EIA units) for sham- and ivermectin-treated groups, respectively. The average ZIKV IgM antibody levels for both groups continuously increased until 15 DPI, with the means (95% CIs) at 83 EIA units (50–117 EIA units) and 91 EIA units (69–113 EIA units) for sham- and ivermectin-treated groups, respectively. Statistical analysis of the effect of ivermectin treatment on ZIKV IgM antibody production revealed no significant differences between the sham- and ivermectin-treated groups at any time point.

### Mosquito ZIKV infectivity and survival results.

**Figure 4.**
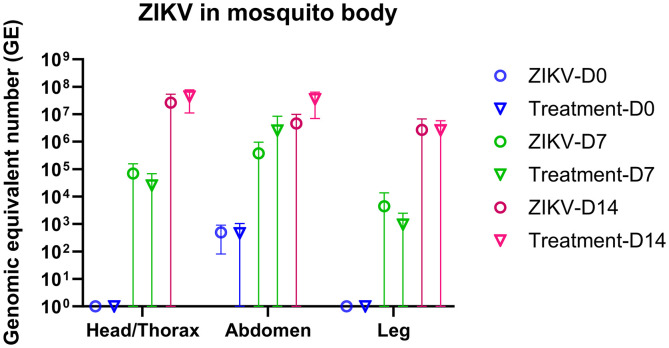
ZIKV RNA levels in the mosquito’s body. Mosquitoes in the ZIKV and ivermectin treatment groups were harvested at 6 hours, 7 days, and 14 days postblood feeding and dissected into three parts, including the head/thorax, abdomen, and leg, before measuring the levels of ZIKV RNA by quantitative reverse transcription polymerase chain reaction. The left *y* axis represents the mean with 95% CI of ZIKV RNA levels in GE units. Blue circles indicate the ZIKV control group at day 6 hours/day 0. Blue triangles indicate the ivermectin treatment group at day 6 hours/day 0. Green circles indicate the ZIKV control group at day 7. Green triangles indicate the ivermectin treatment group at day 7. Red circles indicate the ZIKV control group at day 14. Red triangles indicate the ivermectin treatment group at day 14. The *x* axis represents the different parts of mosquitoes body. GE = genomic equivalent; ZIKV = Zika virus.

To evaluate the effect of ivermectin on ZIKV replication within *Aedes* mosquitoes, mosquitoes were allowed to feed on all NHPs in their assigned groups at 3 days post-ZIKV inoculation and ivermectin administration. ZIKV RNA levels were measured in the head/thorax, abdomen, and leg tissues of these cohorts of mosquitoes at 6 hours, 7 days, and 14 days postfeeding (Figure 4). ZIKV RNA was only detected in the abdomen of mosquitoes at 6 hours postfeeding, regardless of whether they fed on ivermectin-treated NHPs. However, 7 and 14 days after feeding, ZIKV RNA was detectable in the head/thorax, abdomen, and leg tissues of both treatment and control groups. There was no significant difference in ZIKV RNA levels between the two groups at any time point after feeding.

**Figure 5.**
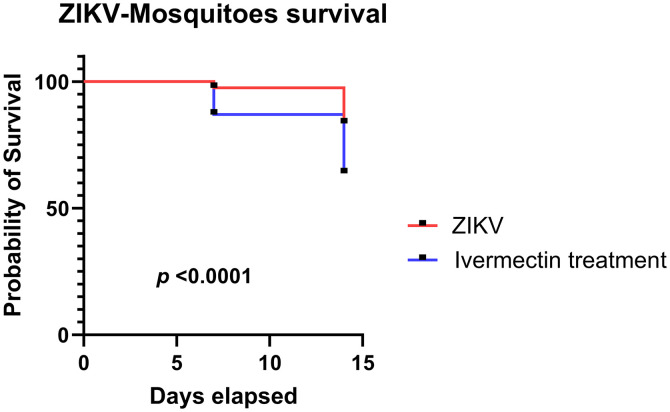
Survival of mosquitoes after feeding on a blood meal containing ivermectin. Mosquitoes in the ZIKV (red line) and ivermectin treatment (blue line) groups were harvested at 6 hours, 7 days, and 14 days postblood feeding. The number of surviving mosquitoes at each time point was collected, and this number was used to calculate the probability of survival using GraphPad Prism v. 9.0.0. The *x* axis represents the days elapsed since the experiment began, and the *y* axis represents the probability of survival. ZIKV = Zika virus.

Furthermore, after the consumption of a blood meal, the number of live/dead mosquitoes in each cohort was recorded at 6 hours, 7 days, and 14 days postfeeding. Compared with mosquitoes that fed on sham-treated NHPs, a significant (*P* <0.0001) decrease in survival was observed at 7 and 14 days postfeeding among cohorts of mosquitos that fed on ivermectin-treated NHPs (Figure 5).

## DISCUSSION

In the in vitro portion of this study, ivermectin significantly reduced the growth rate of both African (MR766) and Asian (Thai isolate SV127/14) strains of ZIKV in mammalian LLC-MK_2_ cells and mosquito C6/36 cells. Notably, the Asian strain exhibited higher sensitivity to ivermectin, with significant viral inhibition observed at lower concentrations (*P* = 0.0023) compared with the African strain in mammalian cell cultures. Furthermore, ivermectin maintained consistently high levels of viral inhibition against the Asian strain but showed diminishing effects against the African strain over time. These differences in susceptibility could be the result of genetic variations between the Asian and African strains. The genetic variation of ZIKV African and Asian genotypes has been reported elsewhere, in which Asian genotypes have approximately 3.5% amino acid variation compared with African genotypes.^35^ These findings diverge somewhat from previous studies; for instance, a study demonstrated that an African strain of ZIKV was significantly inhibited by 1 µM ivermectin treatment, whereas an Asian strain in their study was only significantly inhibited by ivermectin treatments at a concentration of 10 µM.^36^ This indicates the necessity of considering strain variation, treatment strategies, and animal models in evaluating ivermectin’s efficacy against ZIKV.

An NHP model of ZIKV infection was used to investigate whether 3 consecutive days of ivermectin (1.2 mg/kg) prophylaxis could prevent ZIKV infection. The blood concentration of ivermectin considered “therapeutic” is based on reports evaluating its antiparasitic effects and the maximum tolerated dose in rhesus macaques. Despite achieving therapeutic blood level for antiparasitic effects,^34^ ivermectin treatment did not significantly alter ZIKV RNA levels compared with controls. These results suggest that the dosing regimen was insufficient to inhibit ZIKV infection significantly. However, its mechanism of action differs between antiparasitic effects (opening glutamate-gated chloride ion channels) and suggested antiviral effects (inhibition of viral nonstructural proteins). The failure to demonstrate in vivo anti-ZIKV effects may stem from insufficient ivermectin bioavailability, different blood concentrations for its antiparasitic and antiviral effects, and standard formulations that may not effectively inhibit ZIKV. A mouse study similarly found that repeated oral ivermectin administration failed to inhibit ZIKV infection, indicating potential limitations of standard formulations in combating ZIKV.^37^ However, alternative dosing regimens and formulations, such as long-lasting oral tablets,^38^ nanoparticles,^39^ or implants,^40^ might be explored to evaluated ivermectin’s impact on ZIKV infection.

Considering ivermectin’s potential to inhibit ZIKV replication, we examined its impact on the host’s immune response. Anti-ZIKV IgM levels were monitored for 7 DPI, with a final measurement at 15 DPI. Results showed no significant difference in IgM expression between ivermectin-treated and control groups. Although indicating no direct impairment of the NHP immune response, the tested ivermectin regimen failed to reduce ZIKV viremia significantly. Whether a therapeutic ivermectin regimen would indirectly affect the immune response by reducing circulating virus levels remains uncertain.

To evaluate the impact of ivermectin host treatment on onward transmission of ZIKV to mosquitoes, *Ae. aegypti* mosquitoes (the primary mosquito vector of ZIKV) were allowed to feed on ivermectin-treated and sham-treated NHPs at 3 DPI. Groups of mosquitoes were killed at three different time points postinfection (6 hours postinfection, 7 DPI, and 14 DPI), corresponding to three key stages of the viral life cycle in the mosquito: viral infection of the mosquito, viral dissemination from the midgut to other mosquito tissues, and viral transmissibility from the infected mosquito, respectively. This secondary exposure to ivermectin (i.e., consumption of a blood meal from an ivermectin-treated NHP) did not significantly reduce ZIKV prevalence in mosquitoes or RNA levels at any of the three time points. Furthermore, no apparent effect upon viral dissemination throughout the mosquito head/thorax, legs, and abdomen occurred as a result of ivermectin exposure. Taken together, these findings indicate that mosquito secondary exposure to ivermectin in host blood does not directly impair the ZIKV replication cycle in *Ae. aegypti* mosquitos. This is somewhat surprising as an in vivo study found significant reduction in ZIKV prevalence when mosquitoes coingested 10 nM ivermectin.^41^ One possibility could be the method for blood feeding of mosquitoes; they used a glass feeder of blood containing ivermectin, whereas in our study, the mosquitoes received the drug after processing in the NHP body, which seems to be a metabolic form of ivermectin. Secondary exposure to ivermectin did induce a significant but modest reduction in mosquito survival. This was expected as several in vitro studies found an *Ae. aegypti*-lethal concentration that kills 50% of mosquitoes to range from 126 to 601 ng/mL.^42^^–^^44^ However, achieving sustained blood levels in humans at these concentrations long enough to impact ZIKV transmission is unlikely with current formulations as illustrated in the macaque trial, in which they received three high doses of ivermectin (1.2 mg/kg) and only had modest mosquito-lethal effect 72 hours after the last dose. Indeed, human volunteers treated with a standard single oral ivermectin dose (0.2 mg/kg) had no survival effect on *Ae. aegypti* that fed 24 hours posttreatment.^45^

## CONCLUSION

The data generated from this study suggest that the in vitro effect of ivermectin may not necessarily replicate in the in vivo system. Our findings demonstrate that ivermectin can reduce ZIKV replication in both mammalian and mosquito cells in vitro. However, it is crucial to emphasize that this effect was not recapitulated in vivo in the present study. Nevertheless, the secondary transfer of ivermectin from NHP blood to mosquito vectors still impacted mosquito survival rates. However, further refinement of mosquito survival measurements, such as expanding to daily observation, is necessary to construct more robust survival curves. Additionally, it is important to acknowledge that our study did not conduct an in-depth evaluation of various ivermectin dosing regimens. With that said, ivermectin administration did exhibit an insecticidal effect upon mosquito vectors after secondary exposure. Taken together, the results of this study indicate that prophylactic ivermectin may reduce ZIKV transmission indirectly through increased vector mortality after secondary ivermectin exposure. However, further investigation is needed to determine dosing regimens that may realize these effects in vivo.

## Supplemental Materials

10.4269/ajtmh.24-0183 Supplemental Materials
