# Supplementary figures and images for: Ivermectin Inhibits Zika Virus Replication in Vitro But Does Not Prevent Zika Virus Infection in Rhesus Macaques (Macaca mulatta)
Source: Am J Trop Med Hyg. 2024 Dec 17;112(3):648–56. doi: 10.4269/ajtmh.24-0183 (PMC11884300; doi:10.4269/ajtmh.24-0183)

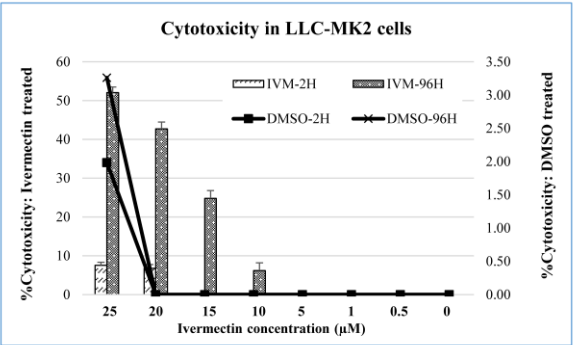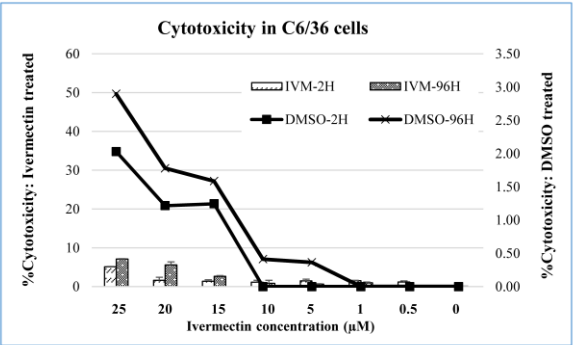

Supplement: Supplemental Materials [file tpmd240183.SD1.pdf]
